# Newly Recognized Pediatric Cases of Typhus Group Rickettsiosis, Houston, Texas, USA

**DOI:** 10.3201/eid2312.170631

**Published:** 2017-12

**Authors:** Timothy Erickson, Juliana da Silva, Melissa S. Nolan, Lucila Marquez, Flor M. Munoz, Kristy O. Murray

**Affiliations:** The University of Texas Health Science Center, Houston, Texas, USA (T. Erickson);; Baylor College of Medicine and Texas Children’s Hospital, Houston (T. Erickson, J. da Silva, M.S. Nolan, L. Marquez, F.M. Munoz, K.O. Murray)

**Keywords:** typhus group rickettsiosis, *Rickettsia typhi*, murine typhus, pediatric infectious diseases, Texas, rickettsial diseases, fleaborne disease, vector-borne infections, United States, bacteria, zoonoses

## Abstract

An increase in typhus group rickettsiosis and an expanding geographic range occurred in Texas, USA, over a decade. Because this illness commonly affects children, we retrospectively examined medical records from 2008–2016 at a large Houston-area pediatric hospital and identified 36 cases. The earliest known cases were diagnosed in 2011.

Typhus group rickettsiosis (TGR) is a vectorborne zoonotic disease most commonly caused by the bacteria *Rickettsia typhi*, the etiologic agent of murine typhus. Since 2003, the annual incidence of TGR cases in Texas has increased and expanded geographically (*1*). Recently, a study conducted in Galveston County found that 7 of 18 persons with acute fever were positive for *R. typhi* (*2*). In the Houston/Harris County metropolitan area, which is adjacent to Galveston County, TGR was first reported to the Texas Department of State Health Services (TXDSHS) in 2007 (*1*). By 2013, 27 cases had been reported to the state. Because the highest attack rate occurs in children 5–19 years of age, we became concerned that children with possible *R. typhi* infection were brought to Texas Children’s Hospital (TCH), a large, 692-bed pediatric hospital system in Houston. To evaluate this recent emergence, we retrospectively searched the hospital diagnostic testing database to identify all TGR-positive patients.

## The Study

We conducted a retrospective review to identify TGR patients seen at TCH from January 1, 2008, through December 31, 2016. We identified cases by searching all laboratory orders for rickettsial panel immunofluorescent antibody (IFA) testing. We determined these patients’ case status by following guidance from TXDSHS (*1*). We defined a confirmed case as 1 of the following: 1) IFA assay titer >1:1024 and a titer for *R. typhi* >2-fold greater than that for *R. rickettsii*; 2) a positive PCR result; or 3) a >4-fold increase in titer between acute and convalescent specimens. We defined a probable case as IFA titer >1:128 and a titer for *R. typhi* >2-fold greater than that for *R. rickettsii*; and a clinically compatible illness involving fever with rash. We defined a suspected case as IFA titer >1:64 and negative titer for *R. rickettsii*; and clinically compatible illness. We abstracted demographic and clinical data on all identified TGR case-patients.

When searching diagnostic laboratory orders for rickettsial IFA, we identified 425 test submissions. On the basis of diagnostic results and clinical compatibility, we identified 36 TGR cases: 18 confirmed, 13 probable, and 5 suspected cases. One case was additionally confirmed *R. typhi*–positive by PCR at the Centers for Disease Control and Prevention (Atlanta, GA, USA). Only 3 case-patients had convalescent specimens collected, and each yielded >4-fold increase in titer, thereby confirming infection.

The case population was predominantly non-Hispanic whites (53%) (Table), which were overrepresented when compared with the proportion of 31% from the Houston/Harris County US Census population for mid-2016 (*3*). Hispanics represented 42% of the patient population, which matched the known census population (42%) The black population was underrepresented (0% of patients vs. 20% Census population) (*3*).

**Table:** Demographic, social, and clinical characteristics of pediatric typhus group rickettsiosis case-patients, Houston, TX, USA, 2008–2016*

Characteristic	All cases, n = 36	Confirmed cases, n = 18	Probable cases, n = 13	Suspected cases, n = 5
Median age (range), y	11 (2–23)	9 (2–23)	9 (4–17)	16 (8–19)
Sex				
M	18 (50)	7 (39)	7 (54)	4 (80)
F	18 (50)	11 (61)	6 (46)	1 (20)
Race/ethnicity				
White	19 (53)	8 (44)	9 (69)	2 (40)
Hispanic	15 (42)	9 (50)	4 (31)	2 (40)
Other or unknown	2(6)	1 (6)	0	1 (20)
Exposures				
Dogs	17 (47)	9 (50)	8 (62)	0
Cats	16 (44)	8 (44)	6 (46)	2 (40)
Opossums	2 (6)	1 (6)	0	1 (20)
Fleas	8 (22)	4 (22)	3 (23)	1 (20)
History of travel				
To endemic area of Texas	4 (11)	4 (22)	0	0
To endemic area outside United States	2 (6)	2 (11)	0	0
To area with no known *Rickettsia typhi*	4 (11)	3 (17)	1 (8)	0
Signs and symptoms				
Fever	35 (97)	17 (94)	13 (100)	5 (100)
Rash	26 (72)	13 (72)	10 (77)	3 (60)
Headache	14 (39)	8 (44)	3 (23)	3 (60)
Malaise	13 (36)	8 (44)	5 (39)	2 (40)
Vomiting	12 (33)	7 (39)	4 (31)	1 (20)
Anorexia	11 (31)	7 (39)	4 (31)	0
Classical triad†	11 (31)	5 (28)	3 (23)	3 (60)
Lymphadenopathy	10 (28)	4 (22)	4 (31)	2 (40)
Abdominal pain	10 (28)	5 (28)	4 (31)	1 (20)
Conjunctivitis	9 (25)	4 (22)	4 (31)	1 (20)
Clinical findings				
Hepatosplenomegaly	6 (19)	4 (22)	2 (15)	1 (20)
Altered mental status	3 (8)	3 (17)	0	0
Elevated aminotransaminases				
Aspartate aminotransaminase	31 (86)	16 (89)	11 (85)	4 (80)
Alanine aminotransaminase	32 (89)	17 (94)	11 (85)	4 (80)
Hypoalbuminemia	11 (31)	6 (33)	3 (23)	2 (40)
Thrombocytopenia	22 (61)	11 (61)	9 (69)	2 (40)
Median days hospitalized (range)	5 (0–14)	6 (1–14)	5 (0–10)	4 (0–5)
PICU admissions	7 (19)	5 (28)	2 (15)	0
Median titer for *R. typhi* (range)	1:1,024 (1:64–16,384)	1:2,048 (1:1024–1:16,384)	1:256 (1:128–1:512)	1:64 (1:64)

*Values are no. (%) except as indicated. A confirmed case was defined by one of the following: 1) immunofluorescent antibody (IFA) assay titer >1:1,024, and a titer for *R. typhi* >2-fold greater than that for *R. rickettsii* to rule out cross-reactivity; 2) PCR positive; or 3) >4-fold increase in titer between acute and convalescent specimens. A probable case was defined as 1) an IFA titer >1:128, and a titer for *R. typhi* >2-fold greater than *R. rickettsii* and 2) clinically compatible illness involving fever with rash. A suspected case was defined as 1) IFA titer >1:64 and negative titer for *R. rickettsii* and 2) clinically compatible illness. †Symptoms were fever, headache, and rash.

Upon review of the medical records, nearly all case-patients (35/36; 97%) were febrile when they sought care (median temperature 103°F) (Table). Eleven (31%) case-patients had the classical triad for typhus of fever, rash, and headache. Other common symptoms included malaise, vomiting, anorexia, abdominal pain, lymphadenopathy, and conjunctivitis. Approximately one fifth had hepatosplenomegaly noted on physical exam. Most case-patients also had elevated transaminases (86%) and thrombocytopenia (61%). A median of 8 days elapsed between symptom onset and arrival at the hospital for medical care and 13 days from symptom onset to defervescence. Whereas the median time from symptom onset to initiation of treatment was 12 days, the median time from initiation of doxycycline treatment to defervescence was only 1 day.

Most case-patients (30/36; 83%) were admitted for hospitalization, with a median length of stay of 5 days. Of those, 7 (19%) required intensive care due to severity of illness. Hispanic patients were significantly more likely to be admitted to the pediatric intensive care unit (86% of these patients were Hispanic; odds ratio 12.7, 95% CI 1.2–612.3; p = 0.027 by 2-tailed Fisher exact test). No deaths occurred.

**Figure 1:**
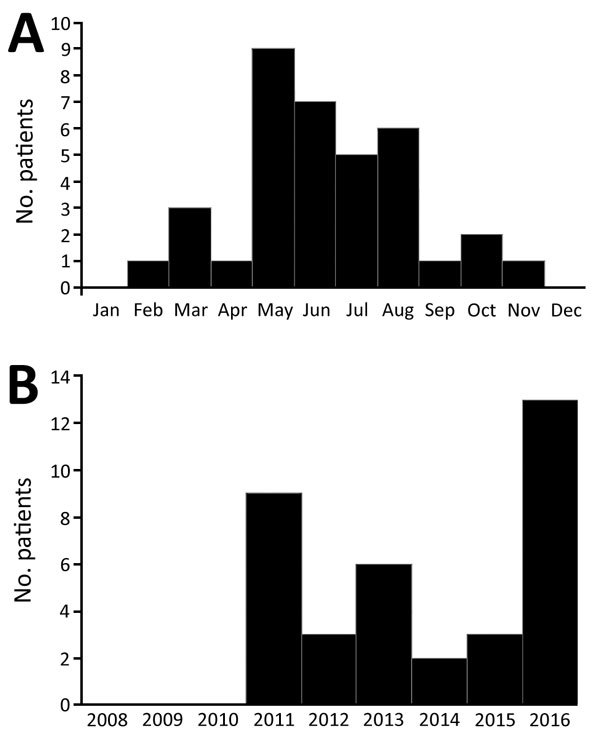
Temporal distribution of *Rickettsia typhi*–positive pediatric case-patients by time of symptom onset, Houston, Texas, USA, 2011–2016. A) By month of symptom onset. B) By year of symptom onset.

All but 2 case-patients had reported animal exposure, and 27 (75%) had reported exposure to either domestic dogs or cats. Two case-patients reported contact with an opossum; 1 of these had no contact with domestic pets. Eight case-patients reported contact with fleas, and all 8 also reported animal contact. Most cases were reported during summer months when fleas are most prevalent (Figure 1, panel A). No TGR cases of were diagnosed in 2008–2010. The year with the highest number of cases was 2016 (Figure 1, panel B).

**Figure 2:**
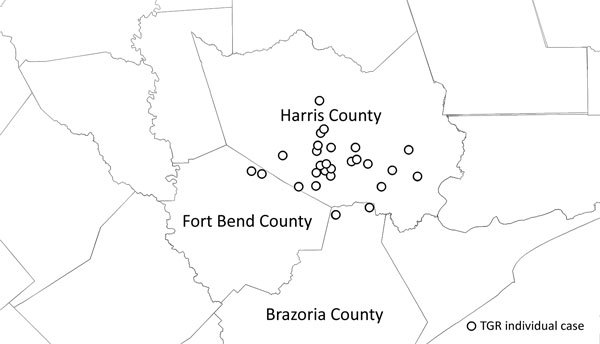
Spatial distribution of *Rickettsia typhi*–positive pediatric case-patients with no history of travel by location of residence around the Houston/Harris County, Texas, USA, metropolitan region.

Thirty (83%) case-patients resided in the Houston metropolitan area; cases were geographically clustered in western Houston (Figure 2). Of these case-patients, 26 (72%) had no history of travel. Three cases were within 70 miles of the Louisiana border. In Louisiana, TGRs are not reportable diseases (*4*).

Recently, we demonstrated evidence of TGR reemergence into new geographic areas of Texas, including Houston/Harris County, the third most populated county in the United States (*1*). Here we present the clinical findings of pediatric patients with TGRs in the Houston metropolitan area, starting in 2011. To track this evidence of emergence, it is critical to raise clinical awareness and encourage testing, diagnosis, and public health reporting of new cases.

Rickettsial infections are reportable in Texas, with cases passively reported by medical care providers. In working with TXDSHS, we cross-referenced our patient list with the public health surveillance database and found only 15 (48%) of the 31 confirmed or probable cases were reported, highlighting a critical gap in passive surveillance. Reporting did improve over time, with 71% (10/14) of 2015–2016 cases reported to TXDSHS, compared with only 29% (5/17) of 2011–2014 cases. Barriers to reporting could be related to low awareness of reporting requirements, complexity of patient care, and the inherent delays in receiving testing results, typically after discharge. Public health surveillance is critical for disease tracking, prevention, and control efforts; therefore, further work is needed to optimize public health reporting of rickettsial infections.

Approximately one third of the pediatric TGR cases we report exhibited the classic triad of fever, headache, and rash, which is considered the hallmark of *R. typhi* infection. Another pediatric study also reported similar findings (*5*). The severity of illness in our patients was remarkable, with 1 in 5 patients requiring intensive care. Early clinical suspicion, diagnosis, and appropriate treatment of suspected rickettsial infections is critical to shorten the duration of illness and prevent serious, life-threatening outcomes.

Our study documents the identification and clinical description of pediatric cases of TGRs in the Houston area. While we believe TGR is emerging locally, it is plausible that prior cases simply went undetected and undiagnosed. A study conducted in 2004 found that 10% of homeless persons in Houston tested seropositive for *R. typhi* (*6*). That study looked only at past exposure, however, so we do not know where those persons acquired the infection. Because studies are lacking in this region regarding the specific reservoirs and vectors responsible for transmission, establishing research in this area is critical.

## Conclusions

The recent emergence of TGRs in Houston poses a public health threat. Our report provides insight into the presentation and epidemiology of disease in a pediatric population. It is still unknown what factors put these children at risk for infection. The sylvatic and domestic transmission cycles of *R. typhi*, including vectors and mammalian reservoirs, require further investigation. Improved physician awareness through reporting of clinical studies and case series will assist in appropriate diagnosis and management of disease throughout Texas and the southern United States.
